# Drought effects on litterfall, wood production and belowground carbon cycling in an Amazon forest: results of a throughfall reduction experiment

**DOI:** 10.1098/rstb.2007.0031

**Published:** 2008-02-11

**Authors:** Paulo M Brando, Daniel C Nepstad, Eric A Davidson, Susan E Trumbore, David Ray, Plínio Camargo

**Affiliations:** 1 Instituto de Pesquisa Ambiental da Amazônia (IPAM)Avenida Rui Barbosa, 136, 68005-080 Santarém, Pará, Brazil; 2 Woods Hole Research Center149 Woods Hole Road, Falmouth, MA 02543, USA; 3 Department of Botany, 214 Bartram Hall, University of FloridaPO Box 118526, Gainesville, FL 32611, USA; 4 School of Natural Resources and Environment, University of FloridaGainesville, FL 32611, USA; 5 Department of Earth System Science, University of CaliforniaIrvine, CA 92697-3100, USA; 6 Centro de Energia Nuclear na Agricultura (CENA)13400-970 Piracicaba, Brazil

**Keywords:** above-ground net primary productivity, wood production, Amazon, drought, litterfall, global change

## Abstract

The Amazon Basin experiences severe droughts that may become more common in the future. Little is known of the effects of such droughts on Amazon forest productivity and carbon allocation. We tested the prediction that severe drought decreases litterfall and wood production but potentially has multiple cancelling effects on belowground production within a 7-year partial throughfall exclusion experiment. We simulated an approximately 35–41% reduction in effective rainfall from 2000 through 2004 in a 1 ha plot and compared forest response with a similar control plot. Wood production was the most sensitive component of above-ground net primary productivity (ANPP) to drought, declining by 13% the first year and up to 62% thereafter. Litterfall declined only in the third year of drought, with a maximum difference of 23% below the control plot. Soil CO_2_ efflux and its ^14^C signature showed no significant treatment response, suggesting similar amounts and sources of belowground production. ANPP was similar between plots in 2000 and declined to a low of 41% below the control plot during the subsequent treatment years, rebounding to only a 10% difference during the first post-treatment year. Live aboveground carbon declined by 32.5 Mg ha^−1^ through the effects of drought on ANPP and tree mortality. Results of this unreplicated, long-term, large-scale ecosystem manipulation experiment demonstrate that multi-year severe drought can substantially reduce Amazon forest carbon stocks.

## 1. Introduction

The fate of approximately 86–140 Pg of carbon contained in the forests of the Amazon (Saatchi *et al*. 2007) will depend, in part, upon the effect of drought episodes on the amount and allocation of forest biomass production. Several lines of evidences suggest that Amazon drought episodes may be more common and more severe in a warming world (Li *et al*. 2006).

One of the most important ways in which severe droughts influence tropical forest carbon stocks is through tree mortality. Droughts associated with El Niño Southern Oscillation (ENSO) events elevated tree mortality in East Kalimantan from a background of 2 to 26% yr^−1^ (Van Nieuwstadt & Sheil 2005), in central Amazonia from 1.1 to 1.9% (Williamson *et al*. 2000), Panama from 2 to 3% (Condit *et al*. 1995), and Sarawak from 0.9 to 4.3–6.4% (Nakagawa *et al*. 2000). (In one Panama forest, ENSO had no effect on tree mortality; Condit *et al*. 2004.) Experimentally induced drought in one hectare of an east-central Amazonian forest permitted the identification of a threshold of declining plant-available soil moisture and cumulative canopy water stress beyond which canopy tree mortality rose from 1.5 to 9% (Nepstad *et al*. 2007).

Responses of net primary productivity (NPP) and its allocation during drought may have important influences on carbon stocks, but they remain poorly understood in moist tropical forests (Houghton 2005). Seasonal and interannual variation in the NPP of moist tropical forests is controlled mostly by changes in light and soil moisture (Clark & Clark 1994). Hence, NPP increases during ENSO events, when reduced cloud cover increases photosynthetically active radiation without provoking soil moisture deficits large enough to inhibit photosynthesis. When soil moisture is in short supply, NPP may decline and its relative allocation among leaves, stems and roots may be affected, but our understanding of these responses is poor. Stem growth is expected to be the most sensitive component of NPP to drought because it is low on the carbon allocation hierarchy (Chapin *et al*. 1990).

The response of root production to drought is less clear, in part owing to substantial methodological challenge of mensuration (Trumbore *et al*. 2006). Soil respiration, which integrates CO_2_ production from all belowground sources, including root respiration and leaf litter decomposition, tends to be lower in the dry season compared with the wet season (Davidson *et al*. 2000, 2004; Saleska *et al*. 2003); this is probably largely due to seasonal variation of heterotrophic respiration in the litter layer. Responses to long-term drought treatments may also include changes in root production.

We conducted a 7-year, partial throughfall exclusion experiment in east-central Amazonia to examine forest responses to a 35–41% effective reduction in annual rainfall. We quantified forest leaf area index (LAI), litterfall, wood production, soil respiration, and the δ^13^C and Δ^14^C composition of CO_2_ emitted from the soil in 1 ha treatment and control plots to test the following predictions:

- wood production is the most responsive component of aboveground NPP (ANPP) to drought, declining rapidly with the onset of drought stress,
- litterfall increases initially through drought-induced leaf shedding, then declines to below non-treatment levels as LAI stabilizes at a lower level, and
- soil CO_2_ efflux is affected by responses of multiple processes of heterotrophic and autotrophic respiration, which could have either accumulating or cancelling effects.

## 2. Material and methods

### (a) Site description and treatment design

The experiment was carried out in the Tapajós National Forest, Pará, Brazil (2.897° S, 54.952° W). Annual precipitation ranges from 1700 to 3000 mm and averages approximately 2000 mm, with a 6-month dry season (July to December) when rainfall rarely exceeds 100 mm per month. Mean annual temperature is 28°C (min 22°C, max 28°C). The soil is deeply weathered Haplustox. The depth to water table at a similar site 12 km away was >100 m, which means that the stocks of soil water available to trees is recharged by rainfall.

The throughfall exclusion experiment was carried out in two structurally and floristically similar 1 ha plots: an ‘exclusion plot’ (treatment) and a ‘control plot’. In the exclusion plot, we diverted approximately 34–40% of annual incoming precipitation (70% of throughfall) during the 6 month wet season from 2000 to 2004. Details of the experimental design can be found in Nepstad *et al*. (2002). Please see Hurlbert (1984) for more information on large-scale unreplicated experiments.

Soil water at 11 m depth was measured monthly in five deep soil shafts per plot between 1999 and 2005 using time domain reflectometry (Jipp *et al*. 1998). Here we present volumetric water content (VWC) for two depth intervals: 0–200 cm (upper soil profile) and 200–1100 cm (deep soil profile).

### (b) Aboveground NPP and tree mortality

Dendrometer bands were installed on trees ≥10 cm dbh (diameter at 1.3 m or above buttresses) and vines >5 cm dbh in the exclusion (342 individuals) and control plots (409 individuals; electronic supplementary material I). Diameter increment was monitored monthly between 2000 and 2005 using an electronic calliper from which relative stem diameter growth was calculated. Wood production at the plot level was calculated by summing biomass estimates for trees and lianas determined using the allometric equations from Chambers *et al*. (2001) and Gerwing & Farias (2000), respectively. We also calculated individual annual stem growth averaged for small (10–20 cm dbh (trees) and 5–20 cm dbh (lianas)) and large (>20 cm dbh) stems. Ingrowth stems were added as they exceeded the lower dbh threshold. Mortality of stems >10 cm dbh in both plots was assessed on an individual basis monthly between 1999 and 2005 and is described in Nepstad *et al*. (2007).

Litterfall was collected bi-weekly from 1999 to 2005 from approximately 64 mesh traps (0.5 m^2^) on a systematic grid in the understorey of both plots. LAI was estimated indirectly monthly at these same points using two LiCor 2000 Plant Canopy Analyzers (LI-COR, 1992) operating in differential mode.

ANPP was calculated as the sum of wood production and litterfall biomass in each year of the study (2000–2005) according to the methodology of Clark *et al*. 2001.

### (c) Soil CO_2_ efflux

A manual dynamic chamber system was used to measure soil CO_2_ efflux on 18 chambers per treatment per date, as described by Davidson *et al*. (2004). Carbon isotope measurements were determined for soil respiration on five occasions from 2000 to 2005 (see electronic supplementary material II).

### (d) Statistical analysis

We used a repeated measures design to test for year-to-year differences between plots for stem growth (using initial dbh as a covariate), LAI, litterfall and soil CO_2_ efflux. Changes in mortality were assessed using a generalized linear model with binomial errors. The relationships among VWC, ANPP and LAI were tested using linear regressions. Further description of the statistical analysis can be found in the electronic supplementary material II.

## 3. Results

### (a) Soil water

**Figure 1:**
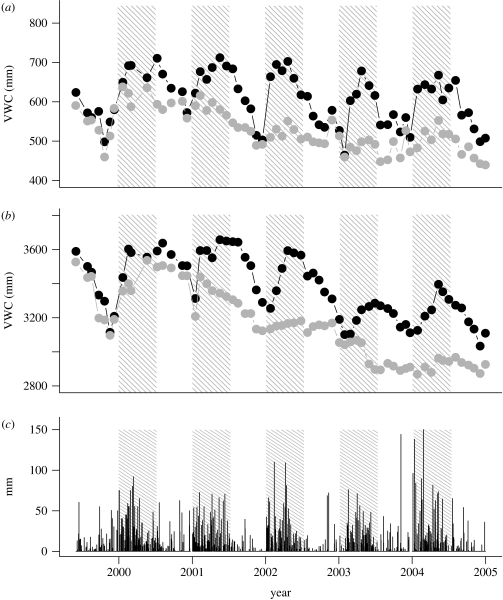
Soil VWC integrated from (*a*) 0 to 200 cm and (*b*) 200 to 1100 cm and (*c*) daily precipitation. Measurements were taken from August 1999 to December 2004. The shaded areas represent the periods of throughfall exclusion. The estimated VWC if the entire soil profile had dried to values equal to the *θ*_r_ residual soil water van Geneuchten parameters for this soil (Belk *et al*. 2007) is 430 and 1950 mm for the 0–200 and 200–1000 cm profiles, respectively. Black circles, control; grey circles, exclusion.

Prior to the initiation of treatment, VWC was 25 and 62 mm lower in the exclusion plot than the control plot for the upper (0–200 cm) and deeper (200–1100 cm) soil profiles, respectively (figure 1*a*,*b*). After three periods of partial throughfall exclusion (2002), VWC was 97 and 272 mm lower in the exclusion plot than the control plot in the upper and deeper soil profiles, respectively. The difference in VWC between plots did not continue to increase in 2003 for two reasons. First, natural precipitation was well below normal that year (electronic supplementary material I), so that soil water was not fully recharged in the control plot. Second, drought-induced mortality in 2003 (see §3*b*) reduced transpiration in the exclusion plot (Cardinot 2007).

### (b) Mortality and necromass

**Figure 2:**
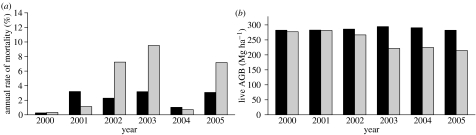
(*a*) Annual per cent of mortality measured on an individual basis for all individuals ≥10 cm dbh in the exclusion and control plots from December 2000 to December 2005. (*b*) Above-ground standing live biomass of all individuals ≥10 cm dbh for the exclusion and control plots during the same period. Black bars, control; grey bars, exclusion.

From 2000 to 2005, the annual mortality rate of individuals higher than or equal to 10 cm dbh was significantly higher in the exclusion plot (5.7% yr^−1^) than in the control plot (2.4% yr^−1^); 2001 was an exception to this general trend (figure 2*a*). The most dramatic treatment effects were observed in 2002 and 2003, when the annual rate of mortality in the exclusion plot reached 7.2 and 9.5% yr^−1^, respectively, as reported in Nepstad *et al*. (2007). Mortality was also significantly higher in the exclusion (7.2 yr^−1^) than the control plot (3.1% yr^−1^) after the termination of the experimental throughfall exclusion, in 2005.

**Figure 3:**
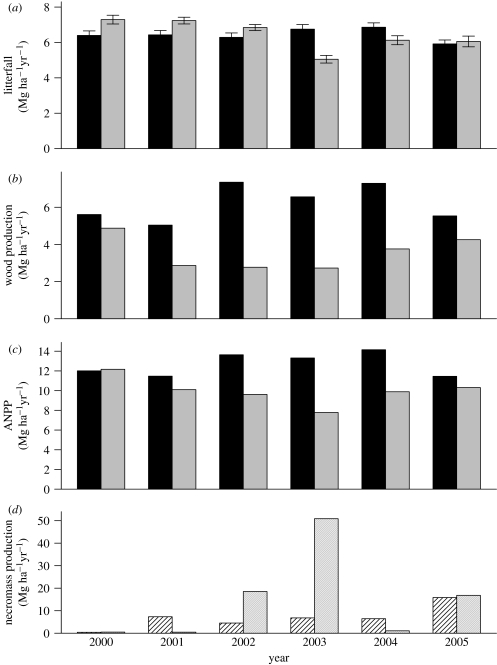
Annual trends in the exclusion and control plots of (*a*) litterfall, (*b*) wood production, (*c*) ANPP and (*d*) necromass production. Measurements were taken from January 2000 to December 2005. Units are Mg ha^−1^ yr^−1^. Error bars (±1 s.e.) are presented only for litterfall. Black bars, biomass control; grey bars, biomass exclusion; shaded bars, necromass control; grey shaded bars, necromass exclusion.

The spike in treatment-induced mortality in 2002 and 2003 led to a 25% reduction in above-ground live biomass (AGLB) in the exclusion plot relative to the control plot (figure 2*b*), a difference that remained stable after the exclusion treatment was terminated in August 2004 and through the end of 2005. From 2000 through 2005, 47 Mg ha^−1^ more necromass [dead biomass] was produced in the exclusion compared with the control plot (figure 3*d*).

### (c) Stem growth and wood production

**Figure 4:**
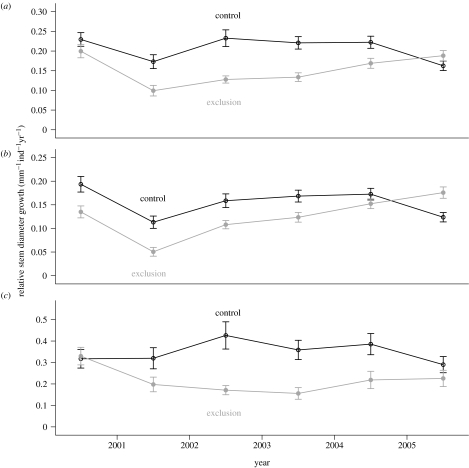
Stem growth of individuals in the exclusion and control plots by different size classes: (*a*) >5 cm dbh (lianas) and >10 cm dbh (trees); (*b*) between 5 (lianas) or 10 (trees) and 20 cm dbh; and (*c*) >20 cm dbh. Error bars indicate ±s.e. (*N* is given by the number of individuals). Measurements were taken from December 1999 to December 2005. Annual stem growth was calculated as the difference in dbh measured between two sampling intervals (December of each year).

The treatment caused a reduction in relative stem diameter growth of large individuals (*p*<0.001) yet had no effect on smaller stems (figure 4*a*–*c*), which were growing less in the exclusion plot than the control plot before the treatment was applied (*p*=0.322; figure 4*b*). Later, following the pulse in the mortality of large trees in 2003, we observed increased stem growth in small individuals in the exclusion plot relative to the control plot (*p*<0.001). During this same period (2004–2005), stem growth was declining in the control plot (figure 4*a*–*c*), perhaps as a lagged response to low annual precipitation in 2003 (figure 1*c*).

Total wood production during the first exclusion period (2000) was 13% lower in the exclusion (4.87 Mg ha^−1^ yr^−1^) than in the control plot (5.61 Mg ha^−1^ yr^−1^). This difference increased over the treatment period to 46, 63 and 58% in 2001, 2002 and 2003, respectively (figure 3*b*). After the drought treatment was removed in 2005, the difference in biomass production between plots declined to 23% due to an increase in individual tree growth (mostly small trees) in the exclusion plot and a decrease in growth in the control plot.

### (d) Litterfall and canopy dynamics

**Figure 5:**
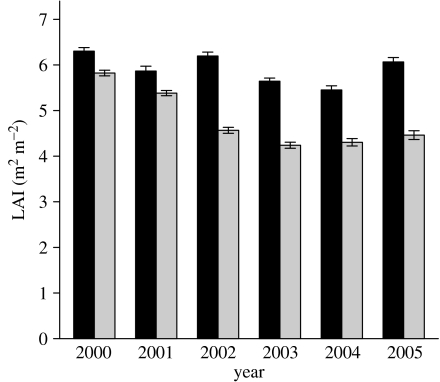
Annual trends in the exclusion and control plots of LAI. Error bars indicate ±s.e. In 2005 LAI is represented by 3 months of measurements. Black bars, control; grey bars, exclusion.

The drought treatment also reduced LAI over time (figure 5; *p*<0.001). Differences between plots in LAI were small during the two first years of the study, followed by a larger drop in 2002 and remaining 21–26% lower than the control plot through 2005.

Litterfall, which was higher in the exclusion plot than in the control plot prior to the initiation of the treatment, declined in the exclusion plot compared with the control plot. However, it was lower in the exclusion plot than in the control plot only in 2003 (23%, *p*<0.001; figure 3*a*). During the last year of the treatment (2004), differences in litterfall between plots were reduced to 10%, and, after the drought stress was removed (2005), litterfall was 2% higher in the exclusion plot than in the control plot, indicating a relative increase in litterfall in the exclusion plot compared with the control plot (*p*<0.001).

### (e) ANPP

ANPP progressively declined in the exclusion plot relative to the control plot from 2000 to 2003: 12, 30 and 41% lower in 2001, 2002 and 2003, respectively (figure 3*c*). In the last year of the treatment (2004) and in 2005, when the exclusion treatment was removed, ANPP differences between the plots were diminished to 30 and 10% of the control, respectively. The average reduction in ANPP from 2000 to 2005 was 21%, corresponding to a cumulative difference in biomass between plots of 17 Mg ha^−1^.

### (f) Correlates of ANPP and LAI

**Figure 6:**
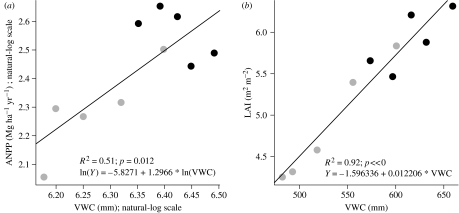
Relationships between annual mean VWC measured in the upper soil profile (0–200 cm) and (*a*) ANPP and (*b*) LAI. Data represent annual means from January 2000 to December 2004. Black circles, control; grey circles, exclusion.

Annual variations in VWC in the upper soil profile (0–200 cm) explained 51% of the variation in ANPP over the 6-year study period (figure 6*a*). Annual average LAI showed a strong positive linear relationship with VWC (*R*^2^: 92%; *p*<0.001; figure 6*b*) and ANPP (*R*^2^: 49%; *p*=0.006; ln(ANPP)=−0.9995+0.8362×ln(LAI)).

### (g) Soil CO_2_ efflux

**Figure 7:**
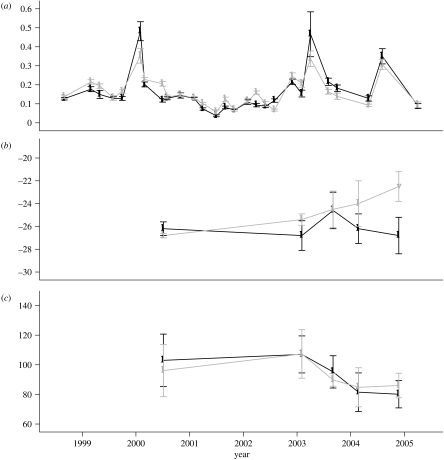
Measurements of surface flux of (*a*) Carbon dioxide (CO_2_ (gC m^−2^ h^−1^)), (*b*) δ^13^C and (*c*) Δ^14^C in the exclusion (grey) and control plots (black). The error bars represent standard errors of the mean.

The effect of the throughfall exclusion treatment on soil CO_2_ efflux was not consistent among years or seasons. Although there was a significant treatment-by-year interaction (*p*<0.001), the treatment-by-year-by-season interaction was not significant (*p*=0.65). When large pulses were measured, which were probably related to recent wetting events, they tended to be higher in the control plot (figure 7*a*). The exclusion plot had somewhat higher CO_2_ efflux rates on most of the other dates during the exclusion treatment years. These differences largely cancelled, resulting in nearly identical estimates of average annual CO_2_ efflux from the two plots during the 5-year exclusion period (12.8±1.0 Mg C ha^−1^ yr^−1^ in the exclusion plot and 12.8±1.3 Mg C ha^−1^ yr^−1^ in the control plot).

The ^13^C signature of the soil CO_2_ efflux revealed a divergence between treatments in 2004, when the ^13^CO_2_ increased (became less negative) in the exclusion plot (figure 7*b*). A similar difference in foliar δ^13^C had been observed for some species in 2002 (J. Ometto & J. Ehleringer 2007, personal communication). We also observed a similar difference in δ^13^CO_2_ measured within the soil profile in 2003 (not shown), which would reflect allocation of fractionated C substrates to root respiration.

There were no differences between treatment plots in ^14^CO_2_ signatures on any date (figure 7*c*), which suggests that the relative age of respired C substrates was not altered by the exclusion treatment. These radiocarbon values are 10–25‰ greater than values for CO_2_ in ambient air sampled at the same time. This indicates that the C being respired is a mixture of modern C substrates with substrates that are 5–40 years old and are more enriched in ^14^C due to the signal of atmospheric testing of nuclear weapons in the 1950s and 1960s (Trumbore *et al*. 2006).

## 4. Discussion

### (a) Response and recovery of ANPP

The most striking result of this study was the differential response of wood production and litterfall to throughfall exclusion. Wood production in the exclusion plot was 13–62% below that of the control plot during each of the five treatment years, while litterfall was substantially lower than the control plot (23%) only during the year of lowest rainfall, in 2003. The allocation of ANPP to wood and litterfall was therefore highly sensitive to soil moisture availability. At the beginning of the experiment (2000), wood and litterfall comprised 40 and 60%, respectively, of the total ANPP, while in the third year of the exclusion (2002), they were 29 and 71% of the ANPP, respectively. This finding provides an important exception to the widely held assumption of drought-insensitive allocation of ANPP between litterfall and wood, as represented in many ecosystem models (e.g. Tian *et al*. 1998; Potter *et al*. 2001; Hirsch *et al*. 2004). These results also run contrary to the positive response of LAI to short-term drought that has been postulated for Amazon forests based upon remotely sensed (MODIS) estimates of LAI (Huete *et al*. 2006; Myneni *et al*. 2007; Saleska *et al*. 2007). If such a positive response is real and widespread, it may be overridden as drought severity grows. However, it is important to remember that our throughfall exclusion treatment did not simulate the higher radiation that is typically associated with lower rainfall.

Litterfall and wood production recovered rapidly following cessation of the drought. Despite the death of 9% of all trees ≥10 cm dbh in 2003, litterfall recovered fully during the first post-treatment year (2005) and wood production, which was 42% of the control plot in 2003, climbed to 77% that of the control plot in 2005. This rapid recovery is partially explained by the positive growth responses observed for residual, surviving trees following the death of neighbouring individuals, perhaps attributable to reduced competition for light and soil moisture resources. LAI declined in the exclusion plot in 2003 and 2004, increasing light availability in the understorey; the difference in VWC between exclusion and control plots also diminished, in part owing to reduced transpiration associated with tree mortality (Cardinot 2007). A second litterfall recovery mechanism was the establishment of fast-growing trees in the exclusion plot understorey (P. Brando & D. Nepstad 2006, unpublished data). Increased soil nutrient availability associated with tree mortality may have also contributed to the accelerated growth of surviving trees and the establishment of fast-growing trees.

The rapid recovery of litterfall in 2004 and after the treatment ended (2005) was, surprisingly, not accompanied by a recovery of LAI. The relationship between LAI and litterfall was similarly unexpected throughout the experiment since litterfall remained high, with a small decline in 2003, despite the sharp, sustained decline of LAI beginning in the third year of the treatment (2002). LAI declined in response to drought with little effect on litterfall either through a lower specific leaf area or through a shortening of leaf longevity or both.

Drought-induced tree mortality and reductions in wood production resulted in a 63 Mg ha^−1^ reduction in AGLB that persisted through the first post-treatment year. The long-term recovery of pre-treatment AGLB will depend upon the balance between wood production and tree mortality. The latter declined to the level of the control plot during the post-treatment year, and could decline further if the drought treatment accelerated the death of trees that were already senescent and prone to die in the near future. Post treatment, tree-level increases in stem diameter growth contributed to an acceleration of wood production which, if it continued, could also reduce the time required to recovery pre-treatment AGLB.

In sum, the experimental drought caused a rapid and drastic reduction in aboveground wood production, while litterfall was less strongly affected. By the third year of the exclusion treatment in 2003, a reduction in litterfall coincided with increased mortality of large trees in the exclusion plot relative to the control plot, suggesting that available soil water had fallen below a minimum threshold required to sustain minimal physiological processes (Nepstad *et al*. 2007). Although the response of plants to soil water depletion may have been initially delayed due to the several coping strategies plants use to avoid or tolerate drought (Oliveira *et al*. 2005; Cardinot 2007), increased mortality and reductions in forest growth resulted in a 24% reduction in ANPP by the end of the experiment, which was highly correlated with a decline in VWC and LAI.

### (b) Below ground carbon cycling

Heterotrophic soil respiration can be inhibited by drought either directly through microbial drought stress or indirectly through substrate limitation (Davidson *et al*. 2006), which is consistent with observations of lower soil CO_2_ efflux rates during the dry season in Amazonian forests (Davidson *et al*. 2000, 2004; Saleska *et al*. 2003). On the other hand, it is also possible that increased allocation of C to fine roots could be an adaptation to explore for deep water resources (Nepstad *et al*. 1994), which might increase belowground inputs of C as a response to drought. The depletion of soil moisture proceeds from the litter and upper mineral soil layers, where root systems are concentrated, to deeper (>8 m in approx. one-third of the Amazon) soil layers (Nepstad *et al*. 1994; Jipp *et al*. 1998). New root growth therefore occurs at progressively deeper soil layers during drought episodes and may be inhibited near the soil surface by the lack of available moisture. These plausible responses that differ in sign may help explain the lack of a consistent and significant treatment effect on soil CO_2_ efflux. Either there were no changes in belowground C cycling processes or the changes that did occur cancelled with respect to CO_2_ production, thus resulting in nearly identical average annual CO_2_ efflux rates.

The isotope data shed some light on these possibilities. First, the ^13^C data demonstrate that C fixed by drought-stressed leaves, which is more enriched (less negative) in ^13^C than in the control plot, was transported to the soil and/or forest floor by litterfall and/or root respiration and root inputs. Hence the heavier ^13^C signatures of C respired in 2004 could reflect the decomposition of enriched-^13^C leaf litter produced 1–2 years previously, a contribution of heavier ^13^C in root respiration reflecting drought stress effects on photosynthetic fractionation, or both. However, the 2-year lag between fractionation of fresh foliage in 2002 and the first detection of that isotopic signal in surface CO_2_ efflux in 2004 suggests that decomposition of aboveground litterfall rather than root respiration (which would reflect the isotopic signature of current year's photosynthate) dominates the surface CO_2_ efflux.

The ^14^C data demonstrate that there was no significant shift in the relative age of respired CO_2_ as a result of the drought treatment. In other words, the relative contributions of respiration from young substrates (root respiration and microbial respiration of exudates and fresh leaf and root litter) and older substrates (root, leaf and wood litter and soil organic matter that has persisted 5 years) did not change appreciably. Rhizosphere respiration typically has a ^14^C signature that matches the atmospheric ^14^CO_2_ of the same year, so that an increase in allocation to roots in an amount necessary to offset a reduction in CO_2_ derived from older litter decomposition in the exclusion plot would have reduced ^14^C values overall. Likewise, had there been significant changes in decomposition rates of soil carbon stocks with higher ^14^C values (leaf and root litter and soil organic matter; Trumbore *et al*. 2006), we should have detected a change in the radiocarbon signature. Hence, we see little evidence of significant changes in belowground C cycling processes induced by the drought treatment. Responses to natural seasonal droughts, which temporarily reduce respiration (Saleska *et al*. 2003), should not be confused with responses to longer-term droughts, which may or may not alter annual rates of C inputs to and turnover times within the soil. These results highlight the danger of extrapolating short-term metabolic responses to longer-term ecosystem level responses that potentially involve changes in C allocation, mortality and growth.

### (c) Carbon stock accumulation and forest recovery

Partial throughfall exclusion induced reduction in AGLB points to the potential for severe and intense droughts predicted for the Amazon basin to increase carbon emissions to the atmosphere. Even mild droughts, such as those induced during the first year of exclusion, can inhibit wood production and, hence, the long-term pattern carbon storage in this forest ecosystem. This finding has important implications regarding reports that many Amazon forests appear to be undergoing net carbon uptake (Grace *et al*. 1995; Malhi *et al*. 1998; Phillips *et al*. 2002; Baker *et al*. 2004)—a finding that has been met with some scepticism (Clark 2002; Saleska *et al*. 2003). Reductions of rainfall in regions of strong seasonal drought, comprising nearly half of the Amazon's forests (Nepstad *et al*. 2004), may reduce wood production, diminishing or reversing the net uptake of carbon from these ecosystems. The long-term effect of the 63 Mg ha^−1^ reduction in AGLB on aboveground carbon stocks will depend upon the complex balance between ABLG regrowth and the rate at which trees are killed by the drought decompose. Many factors influence ABLG re-accumulation, including tree mortality rate, wood production rate and the wood density of the trees that eventually replace those killed by drought stress. Forests recovering from slash-and-burn agriculture in the Venezuelan Amazon required approximately two centuries to recover pre-disturbance carbon stocks in part owing to shifts in species composition (Saldarriaga *et al*. 1988). The decline of LAI and AGLB observed in this study also suggests that the positive feedback between drought and fire may be stronger than previously hypothesized (Nepstad *et al*. 2001), which could have large long-term implications for carbon storage. Hence, even this relatively long-term 5-year experimental manipulation was not long enough to investigate the full effects of climate change on the carbon stocks of Amazonian forests. Nevertheless, we have demonstrated that multi-year drought induced substantial losses of aboveground biomass and productivity.

## Supplementary Material

Supplementary information I Rainfall excluded during the experiment and histogram with diameter size distributions

Supplementary information II Expanded methods
