# Supplementary material for: Drought effects on litterfall, wood production and belowground carbon cycling in an Amazon forest: results of a throughfall reduction experiment
Source: Philos Trans R Soc Lond B Biol Sci. 2008 Feb 11;363(1498):1839–48. doi: 10.1098/rstb.2007.0031 (PMC2374907; doi:10.1098/rstb.2007.0031)
Supplement: Supplementary file 1 [file rstb20070031s04.doc]

**Figure 1** **–** Diameter size class distribution measured in January 2000 of all individuals (lianas: > 5 cm dbh and trees > 10 cm dbh ) in the droughted and control plots.

**Figure 2 –** Annual rainfall (control plot) and effective rainfall (annual rainfall minus water excluded by the plastic panels during the wet season). The partial throughfall exclusion treatment was carried during the six-month wet season each year beginning in January 2000 and ending in August 2004.
