# Supplementary material for: Drought effects on litterfall, wood production and belowground carbon cycling in an Amazon forest: results of a throughfall reduction experiment
Source: Philos Trans R Soc Lond B Biol Sci. 2008 Feb 11;363(1498):1839–48. doi: 10.1098/rstb.2007.0031 (PMC2374907; doi:10.1098/rstb.2007.0031)
Supplement: Supplementary file 2 [file rstb20070031s05.doc]

**Soil CO_2_ efflux**

A manual dynamic chamber system (Davidson et al. 2002) was used to measure soil CO_2_ efflux on 18 chambers per treatment per date, as described by Davidson et al. (2004). Carbon isotope measurements were determined for soil respiration on 5 occasions from 2000 to 2005, using methods described in (Gaudinski et al. 2000). Air in the chamber headspace is first scrubbed to remove ambient CO_2_, then soil respired CO_2_ was trapped on molecular sieve 13X. The CO_2_ was desorbed by heating the molecular sieve to 600 C, then cryogenically purified. A portion was sealed in a pyrex tube and analyzed for stable isotope content, and the remainder was converted to graphite targets using sealed tube zinc reduction methods (Xu et al. in press) Radiocarbon analysis of these graphite targets were performed by accelerator mass spectrometry (AMS) at Lawrence Livermore National Laboratory, Livermore, California (LLNL) and at the W. M. Keck Carbon Cycle AMS facility at UC Irvine, California.

**Statistical Analysis**

***ANPP and its components***

We used a repeated measures design to test differences between the two study plots over time for annual relative stem diameter growth, LAI, and litterfall. The analysis viewed plots (exclusion vs. control) as between-subject effects while year represented the within-subjects effects; all possible interactions were examined. For annual relative stem diameter growth we also included the individuals’ initial dbh as a covariate in the model to minimize size effects on growth. Litterfall and LAI were averaged on an annual basis determined by each sampling point. Thus, degrees of freedom for annual relative stem diameter growth, and LAI and litterfall, were based on number of individuals and number of grid points, respectively. Three models were tested to determine: 1) the initial effects of the treatment [Model 1: within-subject ranging from 2000 to 2003]; 2) the role of mortality on forest “recovery” [Model 2: within-subject ranging from 2003 to 2005], and the overall effects of the treatment [Model 3: within-subject ranging from 2000 to 2005]. To test for annual differences in mortality between plots, we used a generalized linear model with binomial errors. Finally, the relationships among VWC (at both upper and deeper soil profile; only the best fit is presented), ANPP, and LAI were tested using linear models. In some cases it was necessary to log-transform the variables to satisfy assumptions of the analysis. All analyses were carried out using R-Software (R Development Core Team 2007).

***Below Ground Carbon Cycling***

Soil CO_2_ efflux data were not normally distributed, so the data were logarithmically transformed prior to analysis of variance. A repeated measures design was used to test the effects of plot, year, season, and their interactions (please see Davidson et al. 2004). The data were aggregated to a seasonal mean (wet and dry seasons) for each year, from the dry season of 1998 to the wet season of 2005. Plot was a grouping variable; year and season were considered as two repeated trial factors. Because there were pre-treatment differences in CO_2_ fluxes between the two plots (Davidson et al. 2004), the between-subjects test of plot effects is not an adequate test of the throughfall exclusion treatment. Rather, we examined the within-subjects interactions of plot, year, and season across the pretreatment (1998-1999), treatment (2000-2004), and post-treatment (2004-2005) periods to analyze the response to the throughfall exclusion manipulation.
